# Can Lung Imaging Scores and Clinical Variables Predict Severe Course and Fatal Outcome in COVID-19 Pneumonia Patients? A Single-Center Observational Study

**DOI:** 10.3390/life12050735

**Published:** 2022-05-15

**Authors:** Ivan Skopljanac, Mirela Pavicic Ivelja, Danijela Budimir Mrsic, Ognjen Barcot, Irena Jelicic, Josipa Domjanovic, Kresimir Dolic

**Affiliations:** 1 Department of Pulmology, University Hospital of Split, 21000 Split, Croatia; ivan.skopljanac@gmail.com; 2 School of Medicine, University of Split, 21000 Split, Croatia; danijelabudimir@gmail.com (D.B.M.); kdolic79@gmail.com (K.D.); 3 Department of Infectious Diseases, University Hospital of Split, 21000 Split, Croatia; irenajelicic80@gmail.com; 4 University Department of Health Studies, University of Split, 21000 Split, Croatia; 5 Clinical Department of Diagnostic and Interventional Radiology, University Hospital of Split, 21000 Split, Croatia; 6 Department of Surgery, University Hospital of Split, 21000 Split, Croatia; ognjen.barcot@gmail.com; 7 Department of Nephrology, University Hospital of Split, 21000 Split, Croatia; josipa.domjanovic@gmail.com

**Keywords:** lung ultrasound, COVID-19, prognostic, pneumonia, CT, chest X-ray

## Abstract

COVID-19 prediction models mostly consist of combined clinical features, laboratory parameters, and, less often, chest X-ray (CXR) findings. Our main goal was to propose a prediction model involving imaging methods, specifically ultrasound. This was a single-center, retrospective cohort observational study of patients admitted to the University Hospital Split from November 2020 to May 2021. Imaging protocols were based on the assessment of 14 lung zones for both lung ultrasound (LUS) and computed tomography (CT), correlated to a CXR score assessing 6 lung zones. Prediction models for the necessity of mechanical ventilation (MV) or a lethal outcome were developed by combining imaging, biometric, and biochemical parameters. A total of 255 patients with COVID-19 pneumonia were included in the study. Four independent predictors were added to the regression model for the necessity of MV: LUS score, day of the illness, leukocyte count, and cardiovascular disease (χ2 = 29.16, *p* < 0.001). The model accurately classified 89.9% of cases. For the lethal outcome, only two independent predictors contributed to the regression model: LUS score and patient’s age (χ2 = 48.56, *p* < 0.001, 93.2% correctly classified). The predictive model identified four key parameters at patient admission which could predict an adverse outcome.

## 1. Introduction

Since the end of 2019, the world has battled the growing coronavirus disease 2019 (COVID-19) epidemic caused by severe acute respiratory syndrome coronavirus 2 (SARS-CoV-2), with more than 290 million confirmed cases worldwide [1]. The virus is designated as severe acute respiratory syndrome coronavirus 2 (SARS-CoV-2), and the disease is coronavirus disease 2019 (COVID-19). Although the understanding of COVID-19 is evolving, there are still major issues concerning the immune response to SARS-CoV-2, COVID-19 diagnosis, management, prevention, and emerging new variants which make it obvious that SARS-CoV-2 is here to stay and potentially becoming endemic [2,3].

Thoracic imaging, including chest X-ray (CXR) or computed tomography (CT), is essential in the diagnosis of COVID-19 pneumonia. However, previous studies showed that CXR was less sensitive in the detection of COVID-19 lung disease compared to CT, with a reported baseline CXR sensitivity of 69% [4]. The sensitivity of CT is 83–100%, considering the results of reverse transcription-polymerase chain reaction (RT-PCR) tests as the gold standard for diagnosis of COVID-19 [5]. High-resolution CT (HRCT) is the “gold” standard imaging method to evaluate the severity of lung involvement in COVID-19 patients. Despite the above, CXR is still extensively being used in the diagnosis of COVID-19 pneumonia due to its wide availability and relative inexpensiveness. COVID-19 pneumonia changes on CXR are typically ill-defined bilateral alveolar opacities of peripheral distribution. Similarly, the most commonly reported HRCT findings of COVID-19 pneumonia include airspace opacities (ground-glass and/or consolidation), typically subpleural and multilobar involvement, sometimes associated with septal thickening [6]. 

In addition to the aforementioned imaging methods that use ionizing radiation, bedside lung ultrasonography (LUS) is a rapid, non-ionizing, repeatable, and reliable examination technique that is increasingly used by clinicians in the COVID-19 pandemic, especially for hospitalized and critically ill patients reducing the need for transportation. Growing evidence shows that LUS sensitivity is close to that of chest CT and is much higher than that of CXR; its usefulness for the management of patients with COVID-19 pneumonia, from diagnosis to monitoring, follow-up, and even outcome prediction, is also demonstrated [7,8,9,10,11,12]. 

The vast majority of patients with more severe symptoms of the disease have one or more comorbidities, such as obesity and cardiovascular disease, with high mortality among elderly patients [13]. Biochemical and hematological laboratory factors such as lymphopenia, elevated serum ferritin, d-dimer, troponin, C-reactive protein, lactate dehydrogenase, and IL-6 are associated with severe disease, poor prognosis, and increased mortality [14,15,16]. 

Identifying risk factors at hospital admission that can predict the clinical course of the disease would help physicians to provide appropriate and timely therapeutic interventions. The COVID-19 prediction models developed so far have mostly combined clinical features, laboratory parameters, and, less often, CXR findings [17,18,19,20]. To the best of our knowledge, none of the proposed combined prediction models involved lung ultrasound, and that was the main goal of our work.

## 2. Materials and Methods

### 2.1. Study Design

This was a single-center, retrospective cohort observational study.

### 2.2. Inclusion and Exclusion Criteria

**Figure 1:**
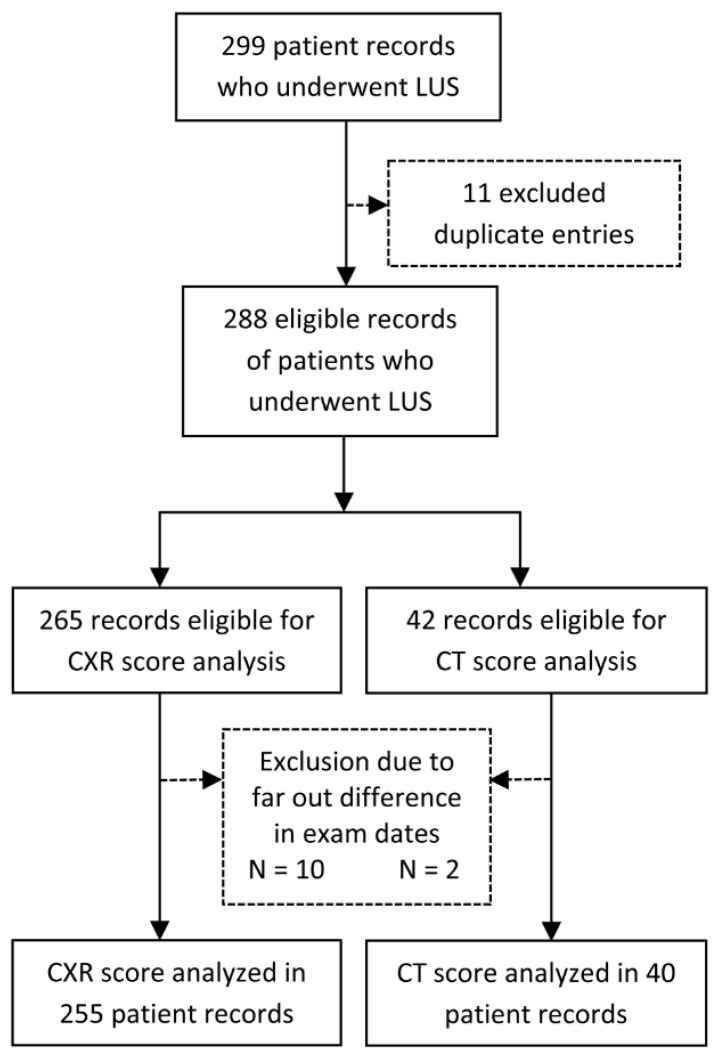
Flowchart of the records included or excluded from the analysis. Legend: CT score—Computerized tomography score; CXR score—chest X-ray score; LUS score—Lung ultrasound score.

The study included a consecutive cohort of patients admitted with the diagnosis of COVID-19 pneumonia in the University Hospital of Split, Croatia, from November 2020 to May 2021, before the COVID-19 vaccine was widely available. Inclusion criteria were WHO diagnostic criteria for pneumonia COVID-19, SARS-CoV-2 infection confirmed by PCR [1], and the existing LUS exam after admission. Exclusion criteria were pulmonary edema associated with heart failure, severe lung emphysema, chronic interstitial lung disease, severe hemodynamic instability and inability to change body position, severe chest deformity, extensive subcutaneous emphysema, any other pulmonary diseases impeding ultrasound image acquisition (i.e., significant pleural effusion, previous pneumonectomy), and an inability to undergo LUS examination (Figure 1).

### 2.3. Outcomes

The study’s primary outcome was the definition of biometric (e.g., age), biochemical (e.g., spO2, LDH), and radiological predictive factors (LUS, CXR, and CT scores) for the necessity of mechanical ventilation (MV) in the treatment of pneumonia or a lethal outcome for the patients.

The secondary outcomes were the comparisons of the reliability and applicability of the three radiological scores assessed.

### 2.4. Data Extraction

Patient demographics, comorbidities, symptoms, laboratory tests, imaging findings, treatment modalities, disease severity, and mortality data were extracted from electronic medical records.

### 2.5. Acquisition Protocol

Lung ultrasound examinations were performed by a trained sonographer (IS) on admission to the hospital using ultrasound equipment (Toshiba Nemio XG Istyle, Tokyo, Japan) with a 1–6 MHz convex transducer. The extent and severity of pulmonary infiltrations were described by a numerically repeatable LUS (Lung Ultrasound Score) coefficient proposed for COVID-19 pneumonia by Soldati et al. [21]. Fourteen areas (three posterior, two lateral, and two anterior for each lung) were examined completely intercostally to cover the widest possible area with a single scan. Changes were scored from 0 to 3. Zero (0) is a regular finding, the existence of a regular and not thickened pleural line, with a sliding sign and the presence of A-lines; 1: denotes an irregular pleural line with some B lines suggesting some loss of aeration; 2: suggests a severe loss of aeration by a broken pleural line and small-to-large consolidated areas with associated areas of white below the consolidated area; 3: is attributed if the scanned area shows large dense consolidations which signify a complete loss of aeration called “white lung” (Table 1). For each patient, the stated scores in all 14 zones were added up (ranging from 0 to 42) to obtain the total LUS score [21]. According to the part of the lung they are positioned in, the 14 areas were grouped in apical, middle, and basal for further statistical analysis. 

CT scans were acquired during hospitalization, as indicated by attending physicians, thus for a limited number of patients. Scanning was acquired on 128 slice multi-slice CT (Philips, Ingenuity Elite). Fourteen CT areas (three posterior, two lateral, and two anterior for each lung) on non-contrast native scans corresponded to fourteen LUS areas. CT findings in each area were classified as follows: 0—no abnormalities, 1—prevalent ground-glass opacities (GGOs), 2—GGOs mixed with consolidations, and 3—prevalent consolidations (Table 1) [22]. For each patient, these scores in all 14 areas were added up (ranging from 0 to 42) to obtain a total CT score.

Chest X-rays were also acquired upon hospital admission, but the CXR score was assessed retrospectively. For CXR scoring, we used the Brixia score [23]. Chest X rays were divided into 3 zones per lung (upper, middle, and lower) in a total of 6 zones: the upper zone extends above the inferior wall of the aortic arch; the mid-zone is the space below the inferior wall of the aortic arch and above the inferior wall of the right inferior pulmonary vein; the lower zone extends below the inferior wall of the right inferior pulmonary vein [23]. Given that, we agreed that anatomically upper CXR zones (zone 1—left, 2—right) approximately corresponded to areas 6 (left) and 3 (right) on the LUS and CT; middle CXR zones (3—left, 4—right) corresponded to areas 2, 10, 14 (left) and 5, 8, 12 (right) on the LUS and CT, and lower CXR zones (5—left and 6—right) corresponded to areas 4, 9, 13 (left) and 1, 7, 11 (right) on the LUS and CT. CXR changes within each zone were scored: 0—no abnormalities, 1—interstitial infiltrates, 2—interstitial and alveolar infiltrates (interstitial predominance), and 3—interstitial and alveolar infiltrates (alveolar predominance) see Table 1. For each patient, the stated scores in all 6 zones were added up (ranging from 0 to 18) to obtain the total CXR score.

### 2.6. Bias

All patient records with an LUS exam were consecutively analyzed without exclusions to minimize selection bias. The only reduction in sample numbers was due to the availability of the other radiological scores, duplicate entries, or based on statistical tests (far out values detection).

The radiological exams were performed by a single clinician (LUS score—IS; CXR score—DBM; CT score—DBM), thus not as a two-person consensus. However, both authors (IS, DBM) are highly experienced in their respective clinical fields. HRCT and LUS exams were blinded and performed without knowledge of laboratory parameters, current treatment, and further involvement in the treatment of the patient. However, the decision to mechanically ventilate the patient was up to the third party without knowledge (blinded) of the LUS score. The CXR and CT scores were assessed upon writing this manuscript with a clinician blinded to any of the observed predictors or clinical outcomes. 

### 2.7. Study Size

This was a sample of consecutive patients collected during the 6-month pandemic peak in Croatia, and the expected enrollment was over 240 cases. We expected the area under the receiver operating curve to be above 0.7 with a ratio of positive outcome cases around 1:10. To accommodate statistical power of 80%, this required a minimum sample of 190 patients.

### 2.8. Statistical Analysis

Descriptive statistics were performed: categorical data were presented by absolute and relative frequencies; continuous data with normal distribution were presented as mean and standard deviation (SE) when highly variable by the median and interquartile range (IQR). The outliers and far-out values were detected by the Tukey method [24]. The normality of the distribution of continuous variables was tested by the Shapiro–Wilk test. The *t*-test was used to compare the means and the Mann–Whitney U test to compare the medians between two groups, while the one-way Chi-square test was used to compare proportions for dichotomous variables. Logistic regression analysis (univariate, multivariate—stepwise method) was used to analyze independent factors associated with the necessity for mechanical ventilation or lethal outcome. The continuous variables included in the models were added to the combined model with their respective slope coefficients as follows: combined score = β1 × var1 + β2 × var2 + β3 × var3 + … + β*n* × var*n*. The receiver operating curve (ROC) was used to determine the optimal threshold, the area under the curve (AUC), specificity, and sensitivity of the tested parameters. Regression analysis was used to describe the relationship between radiological scores. The type I error (alpha) was set to 0.05, and the type II error (beta, statistical power) was set to 80%. The statistical software used for analysis was MedCalc^®^ Statistical Software version 19.6 (MedCalc Software Ltd., Ostend, Belgium; https://www.medcalc.org, accessed on 15 July 2021).

### 2.9. Reporting

We reported the study in line with the STROBE reporting guideline for cohort studies; the STROBE checklist is available in Supplementary File S1.

## 3. Results

### 3.1. Patients and Characteristics

We examined 299 patient records hospitalized in the University Hospital of Split due to COVID-19 from November 2020 to May 2021. Eleven duplicate records or control LUS were excluded, and the rest of the 288 eligible records of patients who underwent LUS were analyzed (Figure 1). Out of 265 records of patients that had a chest X-ray, 10 were excluded due to the dates between LUS and CXR exams being too far apart. Two records out of 42 patients who underwent CT scans were excluded for the same reason. Finally, 255 cases with CXR and LUS and 40 with CT and LUS scores were analyzed.

Patient characteristics between groups not requiring and requiring MV differed only when cardiovascular disease, hemiplegia, or leukemia were present as a negative prognostic factor. Almost the same was noticed in the case of a death outcome (Table 2). Similarly, factors for both the necessity for MV and fatal outcome were the age of the patients and the day of the illness on admission (Table 2). Furthermore, a difference was found in biochemical parameters such as elevated LDH in the group requiring MV, lower spO2, and higher troponin among patients with a fatal outcome. Leukocyte counts were lower in both mechanically ventilated and deceased patients, and respective LUS scores were significantly higher (Table 2).

### 3.2. Predictors of Necessity for MV or a Lethal Outcome

When combined in stepwise multivariate analysis, both CXR and CT scores were excluded from models for either MV or lethal outcome. Only the LUS score was retained in both models as follows (univariate analysis details available in Supplementary File S2).

**Figure 2:**
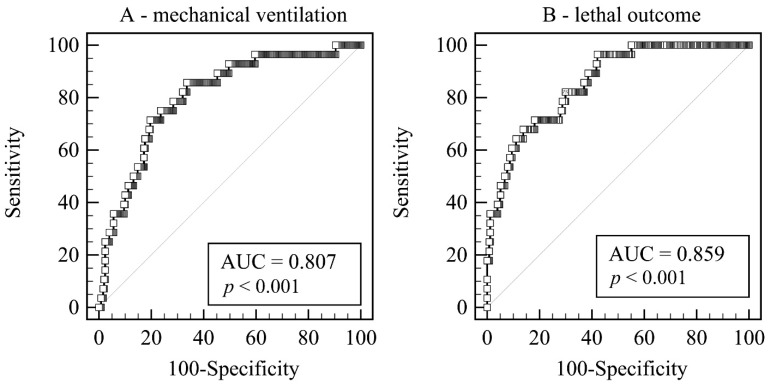
Receiver operating curves of the combined scores. (**A**) Predicting the need for mechanical ventilation. (**B**) Predicting the lethal outcome. Legend: AUC—Area under hierarchical receiving operator curve.

Four independent predictors gave a unique statistically significant contribution to the regression model for the necessity of MV, and these are LUS score, day of the illness at admission, leukocyte count, and presence of cardiovascular disease (χ2 = 29.16, *p* < 0.001). The model accurately classified 89.9% of cases (Table 3, Figure 2A). 

For the lethal outcome, only two independent predictors gave a unique statistically significant contribution to the regression model: LUS score and age of the patient (χ2 = 48.56, *p* < 0.001). The model accurately classified 93.2% of cases (Table 3, Figure 2B).

### 3.3. Relationship between LUS and CXR Scores

The regression model between the CXR score and LUS score demonstrated a strong trend (slope 0.160, 95%CI 0.109 to 0.212, *p* < 0.001); however, there was significant variability around the regression line (R^2^ = 0.128; Supplementary Files S2 and S3).

**Figure 3:**
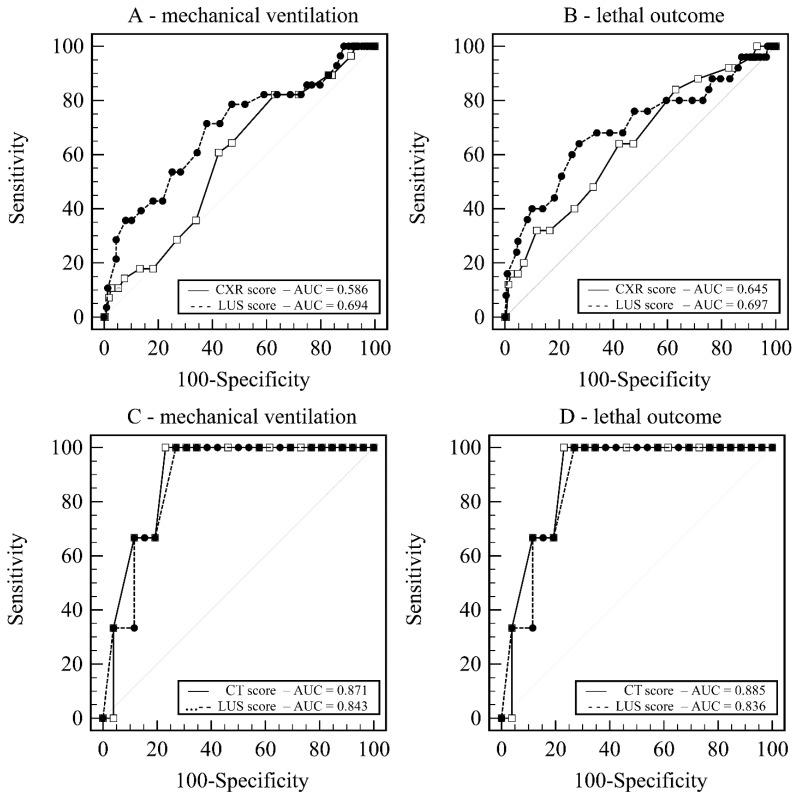
Receiver operating curves. (**A**) LUS score vs. CXR score—prediction of mechanical ventilation. (**B**) LUS score vs. CXR score—prediction of lethal outcome. (**C**) LUS score vs. CT score—prediction of mechanical ventilation. (**D**) LUS score vs. CT score—prediction of lethal outcome. Legend: AUC—Area under hierarchical receiving operator curve; CT score—Computerized tomography score; CXR score—chest X-ray score; LUS score—Lung ultrasound score.

Prediction models for MV based on LUS score (AUC = 0.693 ± 0.058) and CXR score (AUC = 0.586 ± 0.054) showed no significant difference of 0.106, *p* = 0.136 (Figure 3A). 

Additionally, models for death based on LUS score (AUC = 0.697 ± 0.064) and CXR score (AUC = 0.645 ± 0.059) showed no significant difference of 0.052, *p* = 0.449 (Figure 3B).

### 3.4. Relationship between LUS and CT Scores

The regression model between the CT score and LUS score demonstrated a strong trend (slope 0.502, 95%CI 0.292 to 0.711, *p* < 0.0001); however, there was significant variability around the regression line (R2 = 0.396; Supplementary File S2).

Prediction models for MV based on LUS score (AUC = 0.871 ± 0.090) and CT score (AUC = 0.843 ± 0.077) showed no significant difference of 0.023, *p* = 0.819 (Figure 3C). 

Additionally, models for death based on LUS score (AUC = 0.885 ± 0.057) and CT score (AUC = 0.836 ± 0.080) showed no significant difference of 0.049, *p* = 0.582 (Figure 3D).

## 4. Discussion

The results of our study draw us to conclusions defining four independent predictors regarding the regression model for the necessity of MV: LUS score, day of the illness at admission, leukocyte count, and presence of cardiovascular disease. For the lethal outcome, the two independent predictors were the LUS score and the age of the patient. Although LUS’ predictive value was shown in several previous studies, as far as we know, it was not combined with demographics, clinical data, and laboratory parameters, creating prediction models for disease severity.

The average CXR score was not previously shown to be different between the group of patients who needed MV and the group of patients who did not. Although it is a possible prognostic factor for death in the univariate model, the CXR score was dropped from the multivariate model as insignificant in favor of, for example, the patient’s age. Therefore, we can conclude that the CXR score is inferior to the LUS score in the prediction of the need for MV or death.

For both the CT score and the CXR score, we showed a significant correlation with the LUS score by linear regression, although in both cases, there was significant variability. We attributed this to subjectivity in the scoring system based on the decision of the clinician interpreting the finding, the lower sensitivity and specificity of the CXR, and the time interval between CT, LUS, and CXR acquisition, in comparison with other studies that used shorter time delays among them [25]. On the other hand, the narrower CXR score scale (0–18) allowed for a smaller distribution of the total score. It was expected that the relative cutoff value (out of maximal score) would be higher in CXR than in the LUS score, which did not prove to be exact. The cutoff CXR score for the prediction of MV was already at 28% of the maximum score, while for the fatal outcome, it was at 38% and was therefore clinically completely irrelevant for these critical outcomes for the remaining upper ~60% of the score scale. We believe that based on plain CXR, very little can be described, such as the gentle difference of changes in the lung parenchyma essential for the outcomes. The non-specificity of CXR is a possible reason for the relatively low cutoff values obtained.

On the other hand, a much better correlation between LUS score and CT score, with significantly less variability and equal width of the scoring scale, speaks in favor of LUS as an excellent method of choice in monitoring patients with pneumonia; this was confirmed in other studies as well [25,26]. Despite this, previously published studies of predictive models involving radiological imaging have exceptionally included ultrasound more commonly than CT or chest CXR. Huang J. et al. suggested a diagnostic model obtained to distinguish between moderate and severe/critical COVID-19 using CT as a part of the diagnostic procedure. Their model included CT imaging in all patients and even those with mild symptoms [27]. Although CT is the gold standard, of course, the widespread use of such a model, especially in times of intense epidemics, is not possible. The Dutch COVID-19 risk model presented by Schalekamp et al. used similar demographic and laboratory parameters combined with the chest CXR score, which, unlike ours, used four zones in determining the CXR score. As in our study, patients who developed critical illness had leukopenia and higher lactate dehydrogenase levels more often. Contrary to our results, no significant difference was found for the duration of symptoms, although the proportion of patients with a symptom duration of more than 7 days was slightly smaller among those with critical disease [27]. As previously reported, age was a strong predictor of a lethal outcome in our population as well [28].

Multiple studies reported a significant association between elevated leukocyte counts and decreased lymphocyte counts among patients with severe cases of COVID-19 compared with those with mild cases [15,29]. However, in our study population, a decreased leukocyte count was predictive of more severe disease.

Various biochemical markers were researched for their predictive usefulness, for example, LDH, CRP, and D-dimer [30,31,32]. In our study, patients who needed MV had significantly higher LDH values, although LDH did not appear to have predictive power in regression analysis. CRP and D-dimer were not significantly higher in the MV or deceased groups.

In our study, a lower day of illness at hospital admission showed to be a predictive factor for disease severity (MV), as it might suggest a more rapidly evolving and progressive disease in these patients. Similar results concerning onset to hospitalization time were found as a part of the risk nomogram established to predict the incidence of severe or critical COVID-19 in elderly patients in the study by Zeng et al. [20].

The percentage of smokers in the hospitalized population was significantly lower than in the overall population of Croatia, regarding which there is still controversy in the available literature, but most studies still link smoking to a higher risk of severe COVID-19 [32,33,34,35].

The proportion of patients who ended up on MV or died was similar to our previous study [11]. Only the therapeutic options in the patients changed, so we believe that the lower predictability of the LUS score in this study could be related to a modified corticosteroid therapy that the patients received (methylprednisolone 1 mg/kg vs. dexamethasone 6 mg used previously), which might have affected the clinical course and mortality of this disease.

### 4.1. Strengths

Our study was conducted on a large sample of hospitalized patients, and data were analyzed without exclusion by clinical characteristics using only statistically relevant and transparent figures.

### 4.2. Limitations

As mentioned in the bias section of the methods, we did not do a double, independent check of the radiological scores, and this could be a limiting factor. Although we demonstrated a strong association between the LUS score and the CT score, the sample for the CT score was relatively small and did not reach the test strength of 80%.

## 5. Conclusions

The predictive model obtained in our study identified four key parameters at the patient admission to the hospital, LUS score, day of the illness, leukocyte count, and presence of cardiovascular disease, that can predict an adverse COVID outcome. Hopefully, it will help physicians provide appropriate and timely therapeutic interventions.

## Figures and Tables

**Table 1:** Grading of LUS score, CT score, and CXR score.

Score/ Grade	LUS Score	CT Score	CXR Score
0	regular finding: the existence of a regular and not thickened pleural line, with the sliding sign, and the presence of A-lines	no abnormalities	no abnormalities
1	some loss of aeration: irregular pleural line with some B lines	prevalent ground-glass opacities (GGOs)	interstitial infiltrates
2	severe loss of aeration: broken pleural line; small-to-large, consolidated areas with associated areas of white below the consolidated area	GGOs mixed with consolidations	interstitial and alveolar infiltrates (interstitial predominance)
3	complete loss of aeration: scanned area shows large dense consolidations; “white lung”	prevalent consolidations	interstitial and alveolar infiltrates (alveolar predominance)

Acronyms: CT—computerized tomography; CXR—Chest X-ray; LUS—lung ultrasound.

**Table 2:** Patient characteristics according to need for mechanical ventilation or death outcome.

	No MV n/N (%), Median (IQR)	MV Required n/N (%), Median (IQR)	*p* *	No Death n/N (%), Median (IQR)	Death n/N (%), Median (IQR)	*p* *
**Comorbidity/habit**						
Arterial hypertension	132/253 (52.2%)	14/28 (50.0%)	0.827	126/253 (49.8%)	20/28 (71.4%)	0.030
Cardiovascular disease	34/253 (13.4%)	8/28 (28.6%)	0.033	31/253 (12.3%)	11/28 (39.3%)	0.000
COPD	14/253 (5.5%)	1/28 (3.6%)	0.662	14/253 (5.5%)	1/28 (3.6%)	0.662
CVI or TIA	3/253 (1.2%)	0/28 (0.0%)	0.563	3/253 (1.2%)	0/28 (0.0%)	0.563
Dementia	3/253 (1.2%)	0/28 (0.0%)	0.563	1/253 (0.4%)	2/28 (7.1%)	0.001
Diabetes	50/253 (19.8%)	6/28 (21.4%)	0.835	50/253 (19.8%)	6/28 (21.4%)	0.835
Hemiplegia	0/253 (0.0%)	1/28 (3.6%)	0.003	0/253 (0.0%)	1/28 (3.6%)	0.003
Kidney failure	12/253 (4.7%)	1/28 (3.6%)	0.780	10/253 (4.0%)	3/28 (10.7%)	0.107
Leukemia	3/253 (1.2%)	2/28 (7.1%)	0.024	4/253 (1.6%)	1/28 (3.6%)	0.451
Liver failure	7/253 (2.8%)	0/28 (0.0%)	0.374	6/253 (2.4%)	1/28 (3.6%)	0.700
Lymphoma	7/253 (2.8%)	0/28 (0.0%)	0.374	6/253 (2.4%)	1/28 (3.6%)	0.700
Malignancy	25/253 (9.9%)	4/27 (14.8%)	0.425	22/253 (8.7%)	7/28 (25.0%)	0.008
Myocardial infarct	10/253 (4.0%)	3/28 (10.7%)	0.107	10/253 (4.0%)	3/28 (10.7%)	0.107
Peptic ulcer	2/253 (0.8%)	1/28 (3.6%)	0.175	2/253 (0.8%)	1/28 (3.6%)	0.175
Peripheral vascular disease	10/253 (4.0%)	1/28 (3.6%)	0.922	9/253 (3.6%)	2/28 (7.1%)	0.354
Rheumatological disease	8/253 (3.2%)	0/28 (0.0%)	0.341	8/253 (3.2%)	0/28 (0.0%)	0.341
Smoker	33/243 (13.6%)	4/27 (14.8%)	0.860	34/246 (13.8%)	3/24 (12.5%)	0.858
**Biometrics**						
Female gender	68/252 (27.0%)	10/28 (35.7%)	0.952	66/252 (26.2%)	12/28 (42.9%)	0.063
Age (years)	62 (55–70)	69 (62–75)	0.004	62 (54–68)	75 (69–82)	0.000
Weight (kg)	91 (84–103)	94 (82–103)	0.627	91 (84–103)	92 (80–97)	0.388
Height (cm)	180 (173–186)	175 (170–186)	0.356	179 (173–187)	176 (168–180)	0.181
BMI (kg/m^2^)	28.3 (26.1–30.9)	29.4 (27.8–33.1)	0.088	28.4 (26.1–31.1)	28.7 (26.9–32.9)	0.803
Day of the illness	9 (6–12)	6 (5–8)	0.002	9 (6–12)	7 (4–10)	0.035
**Biochemical parameters**						
CRP (mg/L)	76.3 (47.8–136.8)	98 (67.8–146.6)	0.101	79.9 (49.3–136.8)	95.8 (61.4–146.6)	0.416
D–dimer (µg/L)	0.92 (0.61–1.49)	1.05 (0.61–2.20)	0.529	0.88 (0.60–1.48)	1.48 (0.72–2.66)	0.051
LDH (U/L)	351 (282–424)	434 (306–456)	0.043	355 (285–429)	366 (285–448)	0.545
Leukocyte count (10^9^/L)	7.9 (5.7–10.8)	5.7 (4.5–8.7)	0.010	7.9 (5.7–10.6)	5.7 (4.4–10.5)	0.042
Lymphocytes (%)	13.0 (8.4–18.4)	16.3 (10.8–23.9)	0.103	13.0 (8.5–18.4)	16.3 (9.2–23.8)	0.272
Neutrophils (%)	81.2 (74.6–86.9)	75.8 (70.9–83.1)	0.117	81.2 (74.8–86.7)	75.8 (69.4–83.0)	0.123
pO2 (kPa)	7.30 (6.51–7.97)	7.27 (6.18–7.78)	0.599	7.30 (6.50–7.95)	7.20 (6.06–7.97)	0.506
spO2 (%)	91 (88–93)	89 (83–94)	0.151	91 (88–93)	88 (82–93)	0.022
hs-Troponin (ng/L)	9.45 (6.50–15.30)	10.9 (8.0–20.9)	0.216	9.25 (6.55–14.65)	14.6 (8.60–44.10)	0.012
**Radiological scores**						
LUS score	25 (19–31)	31 (26–37)	0.001	25 (18–31)	32 (26–36)	0.000
CXR score	6 (4–10)	8 (6–10)	0.125	6 (4–10)	8 (6–12)	0.016
CT score	22.0 ± 8.0	29.0 ± 2.0	0.146	21.6 ± 7.9	29.0 ± 3.8	0.050

* for dichotomous variables (n/N) one-way classification Chi-squared test was used; for continuous variables without normal distribution, Mann–Whitney U-test was used, and respective *p*-values stated. Acronyms: BMI—body mass index; COPD—chronic obstructive pulmonary disease; CXR score—chest X-ray score; CT score—chest computerized tomography score; IQR—interquartile range; LUS score—lung ultrasound score; MV = 1—mechanical ventilation required, MV = 2—MV not required, death = 0—no death, death = 1—death outcome; SD—standard deviation; TIA—transitory ischemic attack.

**Table 3:** Stepwise multivariate logistic regression according to the necessity of MV or lethal outcome.

**Variable**	**Coefficient**	***p***	**Odds Ratio**	**95% CI**	**Cutoff**	**Sensitivity**	**Specificity**
**Mechanical ventilation**							
LUS score	0.101	<0.001	1.11	1.04–1.17	>27	72.4%	60.8%
Day of illness	−0.131	0.022	0.88	0.78–0.98	≤7	72.4%	64.1%
Leukocyte count	−0.160	0.021	0.85	0.74–0.98	≤6.3	64.3%	66.7%
Cardiovascular disease present	1.019	0.041	2.77	1.04–7.35	positive	28.6%	86.6%
	**Correctly Classified**		**AUC**	**95% CI**	**Cutoff**	**Sensitivity**	**Specificity**
Combined score	88.9%		0.807	0.755–0.851	>0.51	85.7%	66.4%
**Death**	**Coefficient**	***p***	**Odds Ratio**	**95% CI**	**Cutoff**	**Sensitivity**	**Specificity**
Age	0.153	<0.0001	1.17	1.10–1.24	>65	89.3%	65.4%
LUS score	0.088	0.005	1.09	1.03–1.16	>29	69.0%	72.2%
	**Correctly Classified**		**AUC**	**95% CI**	**Cutoff**	**Sensitivity**	**Specificity**
Combined score	93.2%		0.859	0.814–0.901	>9.6	96.5%	57.9%

Acronyms: AUC—area under receiver operating curve; CI—confidence interval; CT score—chest computerized tomography score; LUS score—lung ultrasound score; CXR score—chest X-ray score.

## Data Availability

The datasets used and/or analyzed during the current study are available from the corresponding author on reasonable request.

## Supplementary Materials

The following supporting information can be downloaded at: https://www.mdpi.com/article/10.3390/life12050735/s1, Supplementary File S1—STROBE checklist, Supplementary File S2—Univariate regression of predictors for mechanical ventilation or death, Supplementary File S3—Scatter diagram and regression line between LUS score and CXR score.
