# Supplementary material for: Can Lung Imaging Scores and Clinical Variables Predict Severe Course and Fatal Outcome in COVID-19 Pneumonia Patients? A Single-Center Observational Study
Source: Life (Basel). 2022 May 15;12(5):735. doi: 10.3390/life12050735 (PMC9147733; doi:10.3390/life12050735)
Supplement: Supplementary file 1 [file life-12-00735-s001.zip › life-1705837-supplementary.pdf]

Supplementary File S1

STROBE Statement—checklist of items that should be included in reports of observational studies

	Item No.	Recommendation	Page No.
**Title and abstract**	1	(*a*) Indicate the study's design with a commonly used term in the title or the abstract	2
		(*b*) Provide in the abstract an informative and balanced summary of what was conducted and what was found	2
		**Introduction**	
Background/rationale	2	Explain the scientific background and rationale for the investigation being reported	3
Objectives	3	State specific objectives, including any prespecified hypotheses	4
		**Methods**	
Study design	4	Present key elements of study design early in the paper	5
Setting	5	Describe the setting, locations, and relevant dates, including periods of recruitment, exposure, follow-up, and data collection	5
Participants	6	(*a*) *Cohort study*—Present the eligibility criteria and the sources and methods of selection of participants. Describe methods of follow-up<br>*Case-control study*—Present the eligibility criteria and the sources and methods of case ascertainment and control selection. Present the rationale for the choice of cases and controls<br>*Cross-sectional study*—Present the eligibility criteria and the sources and methods of selection of participants	/<br>/<br>5
		(*b*) *Cohort study*—For matched studies, present matching criteria and the number of exposed and unexposed<br>*Case-control study*—For matched studies, present matching criteria and the number of controls per case	/
Variables	7	Clearly define all outcomes, exposures, predictors, potential confounders, and effect modifiers. Present diagnostic criteria, if applicable	5
Data sources/ measurement	8*	For each variable of interest, present sources of data and details of methods of assessment (measurement). Describe comparability of assessment methods if there is more than one group	5, 6
Bias	9	Describe any efforts to address potential sources of bias	7
Study size	10	Explain how the study size was arrived at	7
Quantitative variables	11	Explain how quantitative variables were handled in the analyses. If applicable, describe which groupings were chosen and why	7-8
Statistical methods	12	(*a*) Describe all statistical methods, including those used to control for confounding	7-8
		(*b*) Describe any methods used to examine subgroups and interactions	7-8
		(*c*) Explain how missing data were addressed	7-8
		(*d*) *Cohort study*—If applicable, explain how loss to follow-up was addressed<br>*Case-control study*—If applicable, explain how the matching of cases and controls was addressed<br>*Cross-sectional study*—If applicable, describe analytical methods taking account of sampling strategy	7-8
		(*e*) Describe any sensitivity analyses	/
		**Results**	

Participants	13*	(a) Report numbers of individuals at each stage of study—e.g., numbers potentially eligible, examined for eligibility, confirmed eligible, included in the study, completing follow-up, and analyzed	10
		(b) Present reasons for non-participation at each stage	10
		(c) Consider the use of a flow diagram	10, Fig1
Descriptive data	14*	(a) Present the characteristics of the study participants (e.g., demographic, clinical, social) and information on exposures and potential confounders	10, Table 1
		(b) Indicate number of participants with missing data for each variable of interest	10
		(c) *Cohort study*—Summarize follow-up time (e.g., average and total amount)	/
Outcome data	15*	*Cohort study*—Report numbers of outcome events or summary measures over time	*10-11*
		Case-control study—Report numbers in each exposure category or summary measures of exposure	/
		Cross-sectional study—Report numbers of outcome events or summary measures	*10-11*
Main results	16	(*a*) Present unadjusted estimates and, if applicable, confounder-adjusted estimates and their precision (e.g., 95% confidence interval). Make clear which confounders were adjusted for and why they were included	*10-11, Table 2, Figures 2,3*
		(*b*) Report category boundaries when continuous variables were categorized	*10-11, Table 2, Figures 2,3*
		(*c*) If relevant, consider translating estimates of relative risk into absolute risk for a meaningful time period	/
Other analyses	17	Report other analyses conducted—e.g., analyses of subgroups and interactions, and sensitivity analyses	*10-11, Table 2, Figures 2,3*
		**Discussion**	
Key results	18	Summarize key results with reference to study objectives	12-13
Limitations	19	Discuss limitations of the study, taking into account sources of potential bias or imprecision. Discuss both the direction and magnitude of any potential bias	14-15
Interpretation	20	Present a cautious overall interpretation of the results considering objectives, limitations, the multiplicity of analyses, results from similar studies, and other relevant evidence	14-15
Generalisability	21	Discuss the generalizability (external validity) of the study results	14-15
**Other information**			
Funding	22	Present the source of funding and the role of the funders for the present study and, if applicable, for the original study on which the present article is based	16

*Give information separately for cases and controls in case–control studies and, if applicable, for exposed and unexposed groups in the cohort and cross-sectional studies.

Note: An Explanation and Elaboration article discusses each checklist item and gives methodological background and published examples of transparent reporting. The STROBE checklist is best used in conjunction with this article (freely available on the Web sites of PLoS Medicine at http://www.plosmedicine.org/, Annals of Internal Medicine at http://www.annals.org/, and Epidemiology at http://www.epidem.com/). Information on the STROBE Initiative is available at www.strobe-statement.org.

Supplementary File S2

Among variables that significantly differed in the group of patients receiving MV vs. the one without the necessity for MV, univariate logistic regression yielded higher age, presence of cardiovascular disease or leukemia, lower leukocyte counts, and higher LUS scores to be possible predictors for MV (Table S1).

For lethal outcome, univariate regression analysis yielded age, presence of cardiovascular disease, malignancy, dementia, arterial hypertension, as well as lower spO2, higher LUS, and CXR scores to be possible predictors (Table S1).

Table S1. Univariate logistic regression according to the necessity of MV or lethal outcome.

Variable	Coefficient	Wald	P	Odds ratio	95% CI	Cases correctly classified	AUC	95% CI
**Mechanical ventilation**								
Age	0.052	7.681	0.006	1.05	1.02–1.09	90.1 %	0.666	0.607 to 0.720
Day of illness	−0.124	6.126	0.013	0.88	0.80–0.97	89.6 %	0.675	0.617 to 0.729
Cardiovascular disease present	0.946	4.286	0.038	2.58	1.05–6.31	90.0 %	0.576	0.516 to 0.634
Leukemia present	1.858	3.941	0.047	6.41	1.02–40.1	90.0 %	0.530	0.470 to 0.589
Hemiplegia present	21.2	0.000	0.998	1.6E+09	/– /	90.4 %	0.518	0.458 to 0.578
Leukocyte count	−0.149	5.173	0.023	0.86	0.76–0.98	90.0 %	0.649	0.590 to 0.705
LDH	0.002	3.656	0.056	1.00	1.00–1.00	89.7 %	0.617	0.556 to 0.675
LUS score	0.091	10.230	0.001	1.10	1.04–1.16	89.8 %	0.691	0.634 to 0.744
CXR score	0.081	2.507	0.113	1.08	0.98–1.20	89.4 %	0.588	0.526 to 0.648
CT score	0.149	2.218	0.137	1.16	0.95–1.41	91.9 %	0.843	0.686 to 0.941
**Death**								
Age	0.139	29.580	<0.0001	1.15	1.09–1.21	90.1 %	0.845	0.798 to 0.885
Day of illness	0.014	1.047	0.306	1.01	0.99–1.04	89.6 %	0.620	0.560 to 0.677
Cardiovascular disease present	1.533	12.608	0.000	4.63	1.99–10.8	90.0 %	0.635	0.576 to 0.692
Malignancy present	1.248	6.487	0.011	3.48	1.33–9.11	90.0 %	0.581	0.521 to 0.640
Dementia present	2.964	5.698	0.017	19.38	1.70 – 221	90.4 %	0.534	0.474 to 0.593
No arterial hypertension	−0.924	4.476	0.034	0.40	0.17–0.93	90.0 %	0.608	0.548 to 0.666
Hemiplegia present	21.2	0.000	0.998	1.6E+09	/	90.4 %	0.518	0.458 to 0.578
spO2 (%)	-0.050	3.919	0.048	0.95	0.91–1.00	89.9 %	0.636	0.576 to 0.694
hs-Troponin	0.013	3.700	0.054	1.01	1.00–1.03	87.5 %	0.673	0.596 to 0.743
Leukocyte count	−0.085	2.127	0.145	0.92	0.82–1.03	90.0 %	0.617	0.558 to 0.675
D-dimers	−0.013	0.092	0.762	0.99	0.91–1.08	90.0 %	0.622	0.557 to 0.683
LUS score	0.106	12.658	0.000	1.11	1.05–1.18	89.8 %	0.724	0.668 to 0.775
CXR score	0.146	6.979	0.008	1.16	1.04–1.29	90.6 %	0.646	0.585 to 0.704
CT score	0.171	3.371	0.066	1.19	0.99–1.42	83.3 %	0.835	0.674 to 0.938

Acronyms: AUC—area under receiver operating curve; CI—confidence interval; CT score—chest computerized tomography score; LUS score—lung ultrasound score; CXR score—chest X-ray score.

Supplementary File S3

Relationship between LUS and CXR scores

The regression model between the CXR score and LUS score demonstrates a strong trend (slope 0.160, 95%CI 0.109 to 0.212, $P < 0.001$); however, there is significant variability around the regression line ($R^2 = 0.128$; Figure S1).

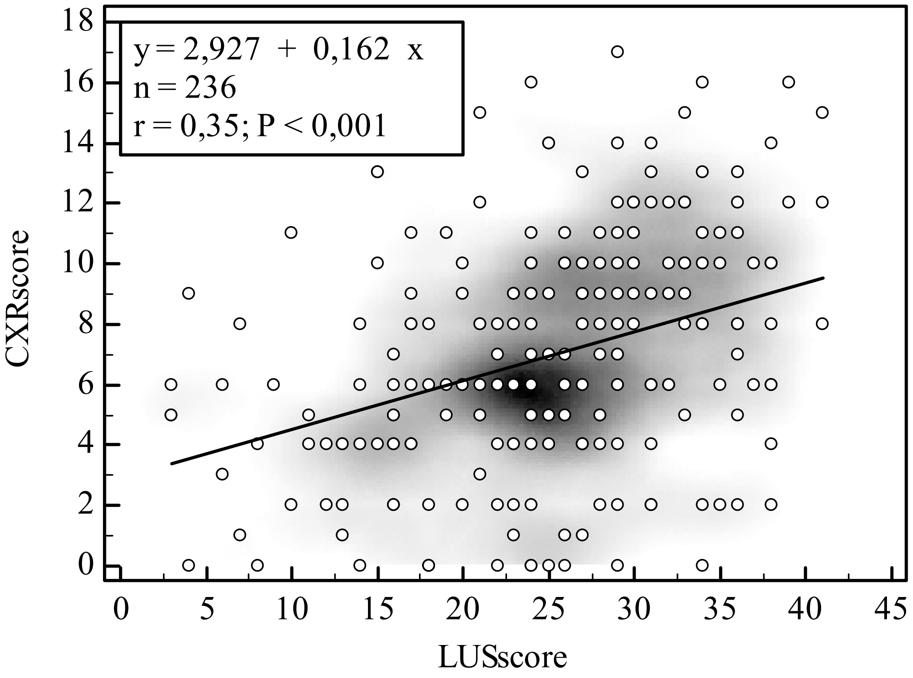

Figure S1. Scatter diagram and regression line between LUS score and CXR score.

The prediction models for MV based on the LUS score (AUC = 0.693 ± 0.058) and CXR score (AUC = 0.586 ± 0.054) show no significant difference of 0.106, P = 0.136 (Figure S2A). Their cutoffs, for best sensitivity and specificity, were at 27 (64% of the maximum score of 42) for the LUS score and at 5 (28% of the maximum score of 18) for the CXR score.

Additionally, models for death based on the LUS score (AUC = 0.697 ± 0.064) and CXR score (AUC = 0.645 ± 0.059) show no significant difference of 0.052, P = 0.449 (Figure S2B). Their cutoffs, for best sensitivity and specificity, were at 29 (69% of the maximum score of 42) for the LUS score and at 7 (38% of the maximum score of 18) for the CXR score.

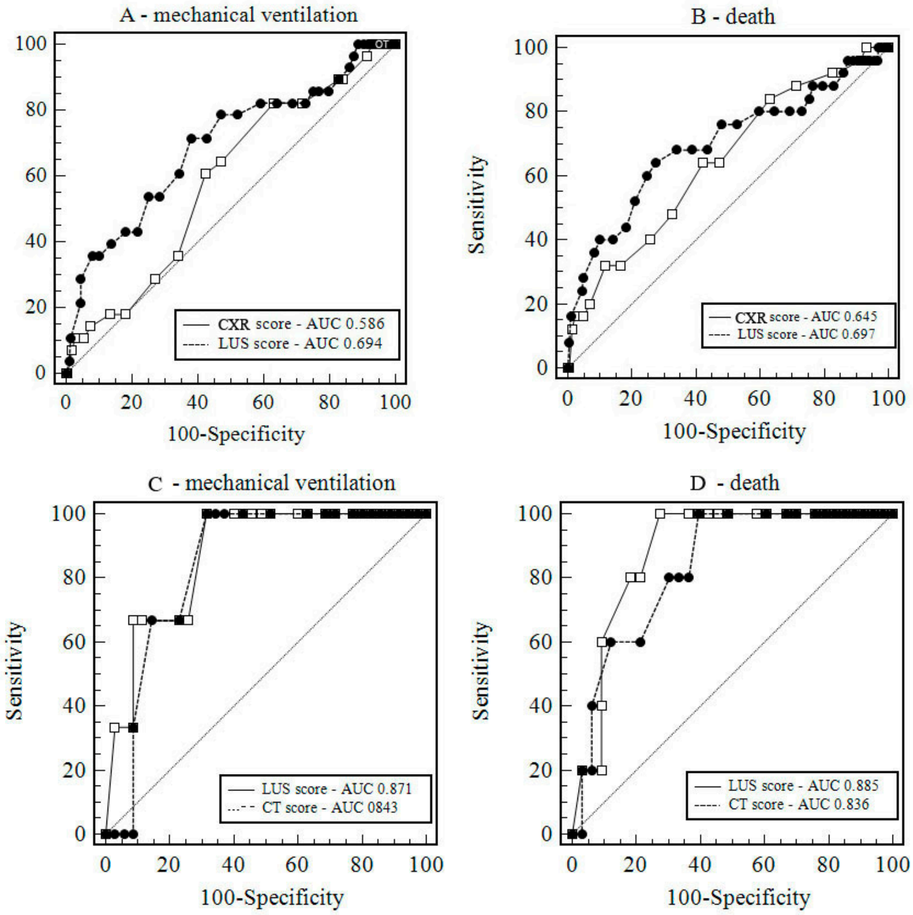

Figure S2. Receiver operating curves of LUS score vs. CXR score for prediction of A – MV and B – death; LUS score vs. CT score for prediction of C – MV and D - death.

Relationship between LUS and CT scores

The regression model between the CT score and LUS score demonstrates a strong trend (slope 0.502, 95%CI 0.292 to 0.711, $P < 0.0001$); however, there is significant variability around the regression line ($R^2 = 0.396$; Figure S3).

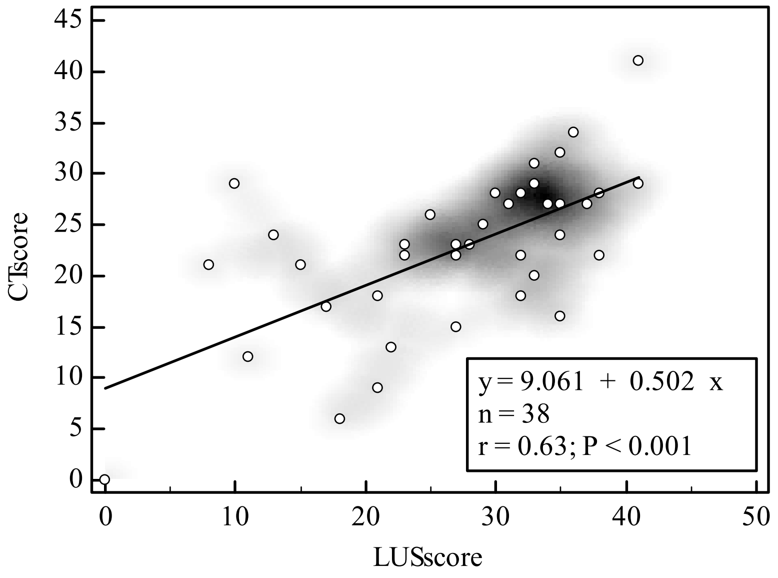

Figure S3. Scatter diagram and regression line between LUS score and CT score.

Prediction models for MV based on the LUS score (AUC = 0.871 ± 0.090) and CT score (AUC = 0.843 ± 0.077) show no significant difference of 0.023, P = 0.819 (Figure S2C). Their cutoffs, for best sensitivity and specificity, were at 32 (76% of the maximum score of 42) for the LUS score and at 26 (62% of the maximum score of 42) for the CT score.

Additionally, models for death based on the LUS score (AUC = 0.885 ± 0.057) and CT score (AUC = 0.836 ± 0.080) show no significant difference of 0.049, P = 0.582 (Figure S2D). Their cutoffs, for best sensitivity and specificity, were at 32 (76% of the maximum score of 42) for the LUS score and at 23 (55% of the maximum score of 42) for the CT score.
